# A hydrogen–deuterium exchange mass spectrometry-based protocol for protein–small molecule interaction analysis

**DOI:** 10.52601/bpr.2023.230006

**Published:** 2023-04-30

**Authors:** Qian Meng, Yuan-Li Song, Chen Zhou, Han He, Naixia Zhang, Hu Zhou

**Affiliations:** 1 Analytical Research Center for Organic and Biological Molecules, State Key Laboratory of Drug Research, State Key Laboratory of Chemical Biology, Shanghai Institute of Materia Medica, Chinese Academy of Sciences, Shanghai 201203, China; 2 University of Chinese Academy of Sciences, Beijing 100049, China; 3 Department of Pharmacology, School of Pharmacy, Shanghai University of Traditional Chinese Medicine, Shanghai 201203, China; 4 School of Pharmaceutical Science and Technology, Hangzhou Institute for Advanced Study, University of Chinese Academy of Sciences, Hangzhou 310024, China

**Keywords:** Hydrogen–deuterium exchange mass spectrometry (HDX-MS), Protein dynamics, Protein–small molecule interactions, Hsp90N

## Abstract

Protein–small molecule interaction is vital in regulating protein functions and controlling various cellular processes. Hydrogen deuterium exchange mass spectrometry (HDX-MS) is a powerful methodology to study protein–small molecule interactions, however, to accurately probe the conformational dynamics of the protein upon small molecule binding, the HDX-MS experimental conditions should be carefully controlled and optimized. Here, we present the detailed continuous-labeling, bottom-up HDX-MS protocol for studying protein–small molecule interactions. We took a side-by-side HDX kinetics comparison of the Hsp90N protein with or without the treatment of small molecules (*i*.*e*., Radicicol, Geldanamycin) for displaying conformational changes induced by molecular interactions between Hsp90N and small molecules. Our sensitive and robust experimental protocol can facilitate the novice to quickly carry out the structural characterization of protein–small molecule interactions.

## INTRODUCTION

Up to now, small-molecule drugs account for over 90% of drugs approved by the United States Food and Drug Administration (FDA) (Zhu *et al.* 2022). Small molecules can alter the biological functions of kinases and receptors by binding into the ATP pocket or through allosteric regulation (Attwood *et al.* 2021; Wu *et al.* 2016). Protein–small molecule interactions are vital in the cell, as they regulate various biological processes including the cell cycle, cell development, and cell signaling (Firestone and Chen 2010; Imming *et al.* 2006; Kim *et al.* 2018). Unveiling protein–small molecule interactions helps us to construct molecular interaction networks and guide drug discovery. However, the study of protein–small molecule interactions lags far behind in total and systematic research (Li *et al*. 2013). There is an urgent need to develop or optimize methodology to provide novel insight into the complicated process.

Hydrogen–deuterium exchange mass spectrometry (HDX-MS) is a powerful technique to study protein structure (Narang *et al.* 2020). HDX-MS monitors the exchange rate of backbone amide hydrogens with deuterium. The HDX rate is directly related to solvent shielding (*i*.*e*., tertiary structure) and intramolecular hydrogen bonding (*i*.*e*., secondary and tertiary structure) (Englander and Kallenbach 1983; Zhang and Smith 1993). Compared to other structure characterization methods (*e*.*g*., X-ray crystallography, nuclear magnetic resonance), HDX-MS enables small quantities of samples (μg) to be measured without mass limitation (Habibi and Thibodeaux 2020; Xiao *et al.* 2018). HDX-MS can also monitor protein conformational dynamics (Huang and Chen 2014). Thus, HDX-MS is a well-suited analytical tool for protein–small molecule interactions (Chalmers *et al.* 2011; Masson *et al.* 2017).

The classical and most widely adopted approach for analyzing HDX kinetics of protein is a continuous-labeling, bottom-up approach, where the target protein is incubated in the deuterated buffer for various time points. The deuterium labeling reaction is quenched by decreasing the pH to 2.5 and the temperature to 0.5 °C. After the quench step, the protein is digested with an acid-active protease (*e*.*g*., pepsin). The peptide mixtures are separated by reversed-phase liquid chromatography and detected by a mass spectrometer (James *et al.* 2022; Liu *et al.* 2020). Although HDX is simple and requires no specialized equipment or chemicals, other than D_2_O, the practice of HDX-MS remains somewhat difficult, not only for newcomers but also for veterans (Hamuro and Coales 2018). The reasons mainly include: (1) back exchange of amide backbone -ND to -NH. Although back exchange continues to receive wide attention, it still averages nearly 30% (Walters *et al.* 2012). (2) spatial resolution (the ability to localize the individual deuterated residues). Although the spatial resolution of the HDX-MS experiment is significantly improved by protease digestion, it is limited by the size (typically 7–15 residues) and the number of proteolysis peptides (Rand *et al.* 2014). (3) data analysis. Although there are many software tools available for automated data analysis and interpretation, it remains a hurdle to analyze overlapping peptides and peptides with low signal intensity (Brown and Wilson 2017).

Here, we presented a detailed and updated protocol of continuous-labeling, bottom-up HDX-MS for protein–small molecule interactions. We assessed the effect of the small molecule (Radicicol) on the higher order structure of the Hsp90N protein. Hsp90 is one of the most important molecular chaperones and its N-terminal domain (Hsp90N) is required for ATP binding and hydrolysis (Pearl and Prodromou 2006; Zhang *et al.* 2015). Radicicol is an antitumor antibiotic which can bind to the Hsp90N protein and disrupt its ATPase activity *in vivo* (Ardestani *et al.* 2022; Li *et al.* 2020; Prodromou *et al.* 2009; Roe *et al.* 1999). The conformation of Hsp90N could be changed as there is a water-mediated network of hydrogen bonds when Radicicol binds to the Hsp90N protein (Khandelwal *et al.* 2018). Based on typical HDX-MS comparison tools (*i*.*e*., butterfly plot, residual plot, deuterium uptake plots, heat map) (Masson *et al.* 2019), our study demonstrated that Radicicol had no significant impact on the Hsp90N's structure except subtle changes of key conserved functional residues. Our HDX-MS protocol can also characterize the modulation of Hsp90N’s higher order structure by Geldanamycin, an Hsp90 inhibitor with a similar action mechanism to Radicicol (Stebbins *et al.* 1997). Overall, the HDX-MS protocol is sensitive, reproducible, and ideally suited to characterize protein–small molecule interactions.

## OVERVIEW OF THE PROTOCOL

**Figure 1:**
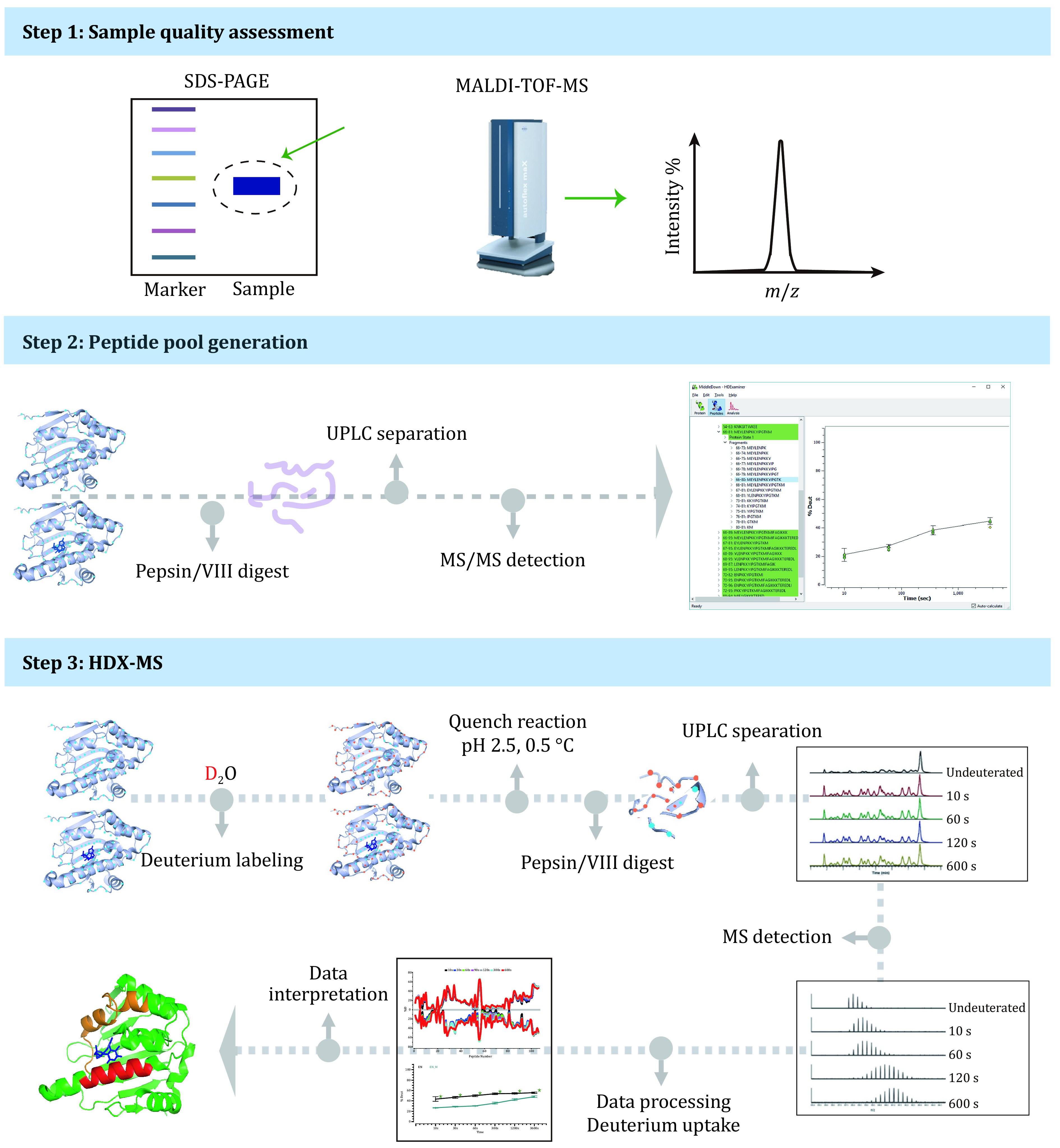
Overview of the HDX-MS protocol

**Figure 2:**
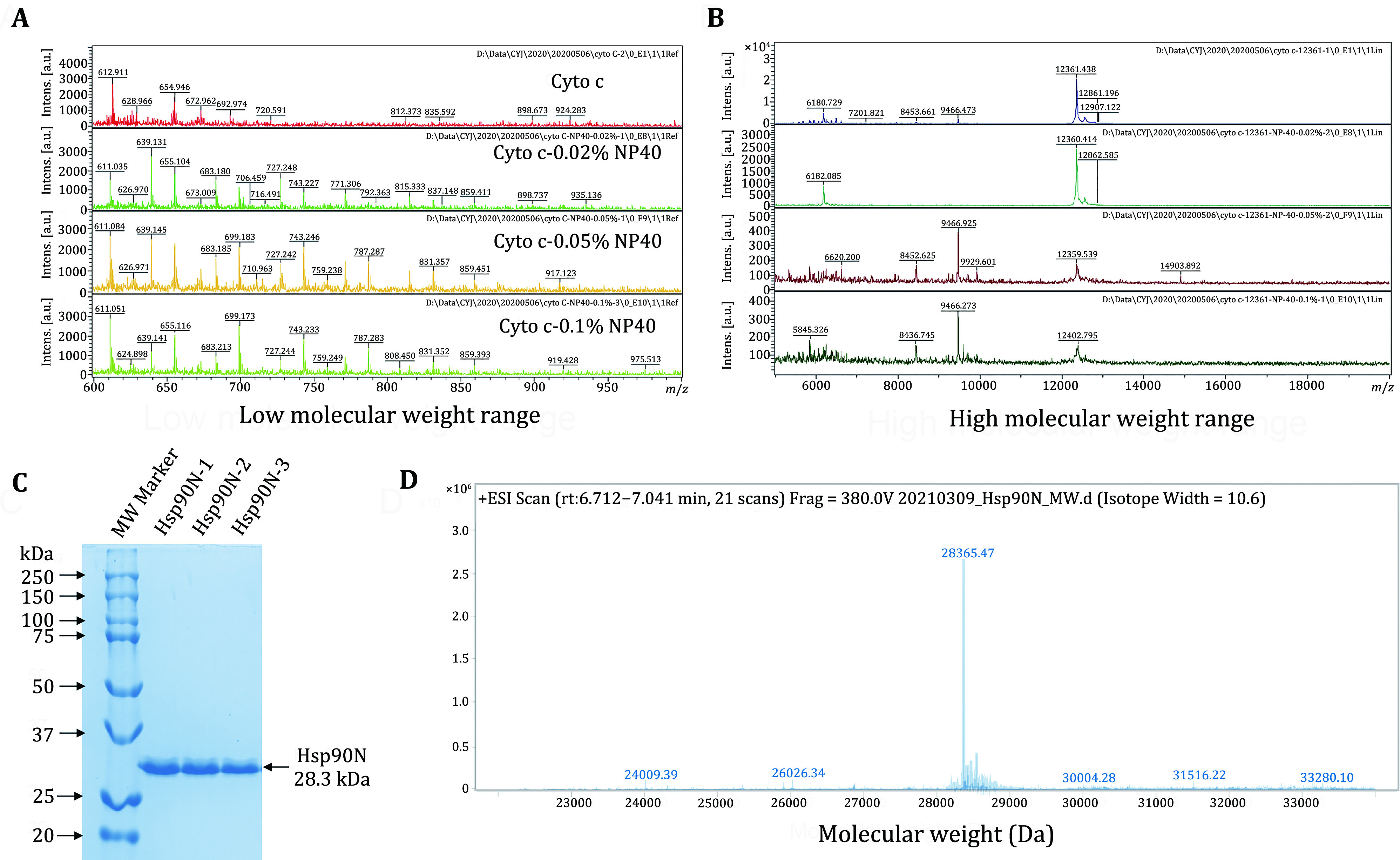
Sample quality assessment. MALDI-TOF mass spectra of cytochrome C in the absence and presence of NP-40. The concentration of NP-40 varies from 0.02% to 0.1%. **A** Low molecular weight range. **B** High molecular weight range. **C** SDS-PAGE analysis of the Hsp90N protein. **D** MALDI-TOF mass spectrum of the Hsp90N protein. The molecular mass of Hsp90N was found to be 28.3 kDa

As shown in Fig. 1, the HDX-MS experiment mainly includes three parts. In the beginning, we should make sure that the target protein is of high quality. Sodium dodecyl sulfate polyacrylamide gel electrophoresis (SDS-PAGE) is a simple and efficient method to confirm molecular weight, protein purity, and post-translational modifications (Laemmli 1970). We should pay special attention to detergents, as they are widely used to isolate and purify proteins, such as membrane proteins and receptor targets (Hothersall *et al.* 2020). However, detergent could damage the performance of chromatographic column and is generally incompatible with electrospray mass spectrometer (ESI-MS) due to signal suppression or enhancement (Furey *et al.* 2013; Loo *et al.* 1996). Matrix-assisted laser desorption/ionization time of flight mass spectrometry (MALDI-TOF-MS) has a higher tolerance to detergent as the samples are detected in crystals (Cheon *et al.* 2021; Sun *et al.* 2023). We can assess the concentration and type of detergents using MALDI-TOF-MS as the mass spectra of samples would display different patterns in mass-to-charge ratios (*m*/*z*) below 1,000 and/or upper 10,000. Taking NP-40 as an example, if there is NP-40 in the samples, the spectra would have a detergent-related diagnostic peak (*i*.*e*., 639.1). The pattern of mass spectra would also vary in response to the detergent concentration (Figs. 2A and 2B).

Then, we should generate a peptide pool via undeuterated samples. The preparation of undeuterated samples is almost identical to that of deuterated samples, except for the deuterium labeling step (replace D_2_O with H_2_O). As the number of peptides in the peptide pool is highly correlated with the comprehensiveness of the HDX profiles of the target protein, we should aim to maximize the protein sequence coverage. We can optimize some critical experimental parameters such as protein concentration, quench buffer composition, protease digestion temperature and time, liquid chromatography (LC) gradient, and mass spectrometer (MS) acquisition parameters by performing pre-experiments.

The bottom-up HDX-MS experiment involves the following steps: deuterium labeling, acid quench, protease digestion, ultra-performance liquid chromatography (UPLC) separation, MS detection, and data analysis. When a native protein is exposed to D_2_O, the deuterium would be incorporated into the protein. However, back exchange (D→H) would exist and can result in significant loss or alteration of deuteration level. The back exchange is highly related to experimental conditions, such as solvent properties (pH, temperature, ionic strength), ion source desolvation temperature, and LC separation (temperature, time) (Keppel *et al.* 2011; Wu *et al.* 2006) As back-exchange is a major hurdle for HDX-MS analysis, we recommend following the pre-optimized experimental workflow to ensure the accuracy and reproducibility of measurement results.

Data analysis is an important topic of the HDX-MS experiment. The ultima goal is to character the conformational dynamics of the target protein induced by small molecule binding. It includes two main parts: generating a peptide pool and analyzing deuterium uptake. To generate a peptide pool, the most commonly applied method is peptide identification and extracting important peptide features such as retention time, mass, charge, and the amino-acid sequence. The deuterium uptake analysis generally involves the following steps: software-based automatic determination of deuterium uptake for each peptide in the peptide pool, manual inspection of the deuterium incorporation profile of all the software-evaluated peptides, and identification of the overall significant deuterium difference peptides. Correlation with the 3D structure of the target protein can also be performed. There are numerous software options available for analyzing HDX-MS data and several reviews related to this topic have been published (Claesen and Burzykowski 2017; Chalmers *et al*. 2011). In this protocol, we adopted BioPharma FinderTM software to identify peptides and HDExamier software for HDX-MS data interpretation (Hamuro *et al.* 2003, 2006; Horn *et al.* 2006).

## MATERIALS

### Reagents

• Tris (Sigma-Aldrich, cat. no. T1503)

• NaCl (Sigma-Aldrich, cat. no. S5886)

• H_2_O (Milli-Q produced)

• D_2_O (Sigma-Aldrich, cat. no. 617385)

• Guanidine Hydrochloride (GnCl, Sigma-Aldrich, cat. no. G3272-500 G)

• Tris(2-carboxyethyl)phosphine (TCEP, Sigma-Aldrich, cat. no. C4706-50 G)

• Citric acid (Sigma-Aldrich, cat. no. 27109)

• Formic Acid (FA, Thermo Fluka, cat. no. 56302)

• Acetonitrile (ACN, Millipore, cat. no. JB085530)

• Radicicol (Sigma-Aldrich, cat. no. R2146-1MG)

• Geldanamycin (MedChem Express, cat. no. HY-15230/CS-0628)

• The Hsp90N protein was kindly provided by Prof. Naixia Zhang’s laboratory at Shanghai Institution of Materia and Medica (Chinese Academy of Sciences)

### Reagent setup

• HDX labeling buffer (20 mmol/L Tris-HCl, 75 mmol/L NaCl, pH 7.4 (H_2_O or D_2_O)) (made fresh monthly)

• HDX quenching buffer (4 mol/L GnCl, 0.2 mol/L TCEP, 100 mmol/L Citric Acid, pH 2.3) (made fresh monthly)

• LC mobile phase A (0.1% FA/H_2_0) (made fresh monthly)

• LC mobile phase B (0.1% FA/ACN)

• Needle cleaning buffer: H_2_O (made fresh weekly)

• Pepsin cleaning buffer: 0.8 mol/L GnCl, pH 2.3 (made fresh monthly)

**[CRITICAL]** The pH of reagents should be carefully checked before the experiment. The pD value is calculated as pD = pH reading + 0.41.

### Equipment

#### Sample preparation

• Centrifuge (Eppendorf, cat.no.5427R)

• Autosampler vials and caps (Thermo Fisher Scientific, cat. no. 6ASV9-S2P)

• Vial inserts (Thermo Fisher Scientific, cat. no. 6PME03C1SP)

• 10 mL Vials & Caps & Seals (Agilent Technologies, cat. no. G6500-88027)

• 15 mL centrifuge tube (Thermo Fisher Scientific, cat. no. 310109003)

• Syringe for injection into HDX manager (LEAP Technologies, cat. no. PAL-SYH-207830, 250 µL; cat. no. PAL-SYH-207826,100 µL, cat. no. PAL-SYH-207813, 10 µL)

• pH-meter with 0.01 pH unit accuracy and 0–14 pH range (WTW inoLab, cat. no. pH7110)

• NanoDrop one Microvolumn UV–Vis Spectrophotometer (Thermo Fisher Scientific, cat. no. 840-317400)

• Timer

• Table-top vortexer (AINGEN, cat. no. OSE-MC8)

#### Chromatographic column

• Protease column: Immoblized protease type XIII/pepsin column (*w*/*w*, 1:2), 2.1 mm × 30 mm, (NovaBioAssays, cat. no. NBA2014005)

• Trap column: Acclaim^TM^ PepMap300 C18 column, 5 µm, 300 Å, 1.0 mm × 15 mm (Thermo Fisher Scientific, cat. no. 163589)

• Analytical column: ACQUITY UPLC Peptide CSH C18 Column, 130 Å, 1.7 µm, 1 mm × 50 mm (Waters, cat. no. 186002344)

#### HDX-MS system

• PAL3 system (LEAP Technologies, the automatic sample handling platform enables temperature control and unattended operation)

• Ultimate NCS-3500 RSLC pump system (Thermo Fisher Scientific)

• Orbitrap Elite MS (Thermo Fisher Scientific)

#### Software

• BioPharma Finder^TM^ (Thermo Fisher Scientific)

• HDExaminer (LEAP Technologies)

• Visualization software: PyMOL (https://pymol.org/2)

• Protein structure download: PDB (https://www.rcsb.org/)

**[CRITICAL]** We recommended testing the HDX-MS system via reference standards pre-experiment to ensure the HDX-MS system is ready for downstream analysis.

## PROCEDURES

### Step1: Sample quality assessment [TIMING 1–2 d]

The Hsp90N protein was analyzed by SDS-PAGE under reducing conditions (β-mercaptoethanol, β-ME). We used 12% separating gel (pH 8.8) and 5% stacking gel (pH 6.8) in parallel with a commercially available pre-stained molecular weight marker (Bio-Rad, cat. no. 161-0396). The Hsp90N protein was also analyzed by Autoflex maX MALDI-TOF-MS (Bruker). Samples were coated with 1 μL 2,5-dihydroxybenzoic acid (DHB) matrix and transferred onto the ground steel target. After drying at room temperature, samples were subjected to MS detection. FlexControl software (Bruker) and FlexAnalysis software (Bruker) were applied in data acquisition and interpretation, respectively.

**[CRITICAL]** Our Hsp90N protein was purified by using a combination of affinity chromatography and size exclusion chromatography (Zhang *et al.* 2015; Zhou *et al.* 2020). To ensure the purity of the target protein, different purification buffers or columns may be needed based on the physiochemical properties of the targeted protein. Detergents are widely used in protein purification. However, detergents may interfere with MS analysis due to ion suppression (Furey *et al.* 2013). It is recommended low concentrations of nonionic detergent (*e*.*g*., dodecyl maltoside) if you must add detergents during the protein purification steps. Before the HDX-MS experiment, samples should also be assessed for the presence of detergents as good sample quality is a prerequisite for downstream HDX-MS experiment.

### Step2: Peptide pool generation [TIMING 2–3 d]

#### Sample preparation

The PAL3 System was used for automatic and efficient sample preparation. Specifically, 3.0 μL Hsp90N protein (15 μmol/L) was automatically dispensed into a vial and diluted 10-fold with the HDX labeling buffer (20 mmol/L Tris-HCl, 75 mmol/L NaCl, pH 7.4 (H2O)) at 10 °C in the HDX Holder 1. The sample was transferred to a vial in the HDX Holder 2 at 0.5 °C and quenched by adding an equal volume of quench buffer (4 mol/L GnCl, 0.5 mol/L TCEP, 100 mmol/L Citric Acid, pH 2.3) for 1 min before online digestion (supplementary Fig. S1A). The complete sample preparation procedures were repeated three times.

#### Online digestion and LC separation

The quenched sample was immediately injected into an immobilized protease type XIII/pepsin column for 3 min at a flow rate of 45 μL/min and delivered by the loading pump of the Ultimate NCS-3500 RSCL-pump system. The digested peptides were trapped and desalted using trap column (Acclaim^TM^ PepMap300 C18 column). Peptides were separated and eluted by analytical column (ACQUITY UPLC Peptide CSH C18 Column) at the flow rate of 45 μL/min using the NC pump of Ultimate NCS-3500 RSCL-pump system. The LC gradient was 0–2% over 4 min, 2%–10% over 0.1 min, 10%–28% over 9.9 min, 28%–80% over 0.1 min, 80% over 1.9 min, 80%–2% over 0.1 min, and 2% over 1.9 min. The enzyme digestion process and the entire LC separation process were performed at 4 °C and 0.5 °C in the stand-alone temperature-controlled pepsin column compartment and temperature-controlled HPLC of the PAL3 system, respectively.

**[CRITICAL]** The UPLC system for HDX-MS was relatively complicated as it contained a Loading pump and NC pump (supplementary Fig. S1B). The general workflow was as follows:

1. Digestion mode. The deuterium protein was injected into the sample loop using the injection valve and digested by an online protease column. The digested fragments were then collected on the trap column.

2. Elution mode. After desalting the peptides using the loading pump, the purified peptides were separated by an analytical column with NC pump and analyzed by MS.

3. Wash mode. The protease column and analytical column would be equilibrated and back-flush with the Loading pump and NC pump, respectively.

The LC gradient is 0–2% over 4 min corresponding to the digestion process (3 min) and desalting process (1 min). The LC gradient corresponding to the elution process with 2%–80% over 12 min can be optimized for the target protein to acquire good chromatography separation.

#### Tandem MS detection

Data were acquired using an Orbitrap Elite MS. Peptides were detected using full-scan mass analysis, from *m*/*z* 350 to 1,500 at a resolution of 60,000 at 400 *m*/*z*, with data-dependent MS/MS analyses trigged by the ten most abundant ions from the parent mass list of predicted peptides. Peptides with single or unassigned charge states were rejected. Higher-energy collision dissociation (HCD) with a collision energy of 35 was used as the fragmentation technique. Dynamic exclusion duration of 30 s and isolation width of 2 Da were enabled.

#### Data analysis

We adopt the peptide mapping module of BioPharma Finder^TM^ software to analyze the tandem MS data. Software parameters were as follows: protein sequence: Hsp90N protein, protease: XIII/pepsin (N-Term: FIMYWV, C-Term: CDEFLMTWY, specificity: high), sidechain N deamidation, and MW oxidation. The experimental result would be filtered with the “MS2” ID Type and “≥80” Confidence score. The sequence, charge state, and retention time of each confirmed peptide were exported to Excel as an "Hsp90N.xls" file for HDX-MS data analysis.

**[?TROUBLESHOOTING (spatial resolution)]** The spatial resolution meant the ability to localize individual deuterated residues and can be obtained via the deuteration level difference of two peptides sharing overlapping regions. To achieve a high spatial resolution, protein digestion needs to be efficient. There were two critical steps:

1. Quench buffer composition. When necessary, protein unfolding can be promoted by the inclusion of low concentrations of denaturants (*e*.*g*., urea, GnCl) or reducing agents (*e*.*g*., TECP).

2. Enzyme digestion. Pepsin is the most widely used protease as it remained a high level of digestion efficiency at low pH and can generate peptides of reasonable length (typically 10–20 amino acids) (Zhang *et al.* 2010). Combining with other proteases like protease type XIII (*i*.*e*., aspergillopepsin or Fungal XIII) can increase the number of obtained overlapping peptides. Longer digestion time and higher digestion temperature also increase digestion efficiency but at the expense of back exchange.

### Step3: HDX-MS [TIMING 9–10 d]

#### Sample preparation

The Hsp90N protein was incubated with Radicicol or Geldanamycin or DMSO (with equal volume to small molecules as control) at 1 to 10 molar ratios on ice. Radicicol-free and Radicicol-bound Hsp90N proteins were incubated with HDX labeling buffer (20 mmol/L Tris-HCl, 75 mmol/L NaCl, pH 7.4 (D_2_O)) for a series of time points (*i*.*e*., 0, 10, 30, 60, 90, 120, 300 and 600 s) at 10 °C. The reaction was quenched by cold acidic quench solution (4 mol/L GnCl, 0.2 mol/L TCEP, 100 mmol/L Citric Acid, pH 2.3) at 0.5 °C. The labeling and quench procedures were repeated three times.

**[CRITICAL]**

1. Deuterium labeling time. Labeling time typically ranges from seconds to hours depending on the protein’s stability. For intrinsically disordered proteins, only a few seconds can discriminate the deuteration difference. For extremely stable proteins, the labeling time could be extended to days. The minimum requirement of the time point numbers is typically four, spanning from seconds to hours (Narang *et al.* 2020).

2. Sample preparation temperature and pH. The HDX rate is highly dependent on temperature and would increases 10-fold with every 22 °C (James *et al.* 2022). The HDX reaction is more efficiently base-catalyzed than acid-catalyzed and reaches a minimum at pH 2.5–3 (Brown and Wilson 2017). We should carefully optimize these parameters to make a balance between on-exchange labeling (deuterium increase) and off-exchange labeling (deuterium loss).

3. Technical replicates. There should be at least three repeated labeling reaction experiments for each time point to allow a reasonable estimate of the data variance.

4. Protein to small molecule ratio. The ratio of protein to small molecules should be adjusted according to their physicochemical properties. It is recommended a molar ratio of 1 to 10 for the first time.

5. The concentration of D_2_O. To ensure greater deuterium incorporation, experiments are recommended to conduct at higher concentrations of D_2_O (80%−90%).

#### Online digestion and LC separation

The experimental procedures were in accordance with “Peptide pool generation”.

**[CRITICAL]**

1. Back exchange primarily occurs during chromatographic separation and varies with the length of the LC process. A short separation time (usually 5−15 min) (Zhang *et al.* 2009) at 0.5 °C and/or UPLC (Wu *et al.* 2006) with superior resolution, speed, and sensitivity can improve the peptide separation, meanwhile, control back exchange.

2. LC carry-over. Online protease column digestion may retain some sticky peptides that distort HDX rate measurement. It is recommended an extra wash of the protease column and back-flush of the trap column as well as the analytical column if the isotope envelopes of the peptides become bimodal distributions (Hamuro and Coales 2018; Majumdar *et al.* 2012).

#### MS detection

Data were acquired using the Orbitrap Elite MS. Peptides were detected using full-scan mass analysis, from *m*/*z* 350 to 1,500 at a resolution of 60,000. Xcalibur software (Thermo Fisher Scientific) was applied in data acquisition.

**[CRITICAL]** It is recommended using high-resolution mass spectrometers (HRMS) with high mass accuracy as there are partially overlapping isotopic peak clusters for the continuous labeling experiment.

#### Data analysis [TIMING 4–5 d]

HDExamier software was adopted for HDX-MS data interpretation. HDExamier software uses an isotope cluster modeling algorithm and computes the geometric centroid of each peptide’s isotope cluster. A peptide’s deuteration level at a given time point is computed by comparing the centroid of the deuterated isotope cluster with its undeuterated counterpart.

The detailed procedures of HDExamier software were as follows:

1. Create a new project “Hsp90N–small molecule”, import protein sequence “Hsp90N”, and set up protein comparison states as “Hsp90N–small molecule” and “Hsp90N”.

2. Create a peptide pool by importing the "Hsp90N.xls file”.

3. Import HDX-MS data files and carefully check the experiment conditions (*e*.*g*., un-deuterated, partially deuterated, fully deuterated).

Some important parameters of HDExamier software were as follows:

1. Deuteration level

(A) The full deuteration level was 89.1% (the percentage of D_2_O in the solution, *v*/*v*).

(B) Assume the first two residues of a peptide cannot hold deuterium.

2. Retention time (RT). Although the HDX-MS experiment was all run under the same chromatographic conditions, they could have some unusual retention time drift. HDExaminer software automatically adjusted retention time via a feature alignment algorithm. The software parameters were as follows:

(A) The non-Deuterated RT window must be within 0.50 min of the expected RT.

(B) The deuterated RT window must be within 0.50 min of the non-deuterated RT window.

(C) Maximum RT envelope 0.50 min.

After the automatic search of HDExaminer software, it is necessary to manually inspect all peptides and their deuterium incorporation profiles. Experimental procedures mainly included:

1. Delete the peptides which are color-coded red (low confidence).

2. Manually check and adjust the retention time or *m*/*z* range of individual peptides. We would shift right-drag a range on the spectrum.

The HDX-MS results mainly included:

1. HDX heatmap to analyze protein deuteration behavior.

2. Butterfly plot and residual plot to show the difference between the two protein states you’ve selected.

3. Peptide’s uptake plot to analyze each peptide’s deuteration behavior over time.

**[CRITICAL]**

1. As the concentration of D_2_O (%, *v*/*v*) presented during the labeling reaction equals to the full deuteration level (maximally labeled level), it must be precisely maintained and clearly reported.

2. The confidence level of a peptide is calculated using various factors, including score, signal-to-noise, and how well the theoretical isotope cluster matches the actual data. It can serve as an indicator for downstream analysis.

3. HDExaminer software would draw confidence interval bars for each peptide by calculating a student’s *t*-distribution using all replicates and charge states available.

**[?TROUBLESHOOTING (data analysis)]**

Although HDX-MS is a well-suited technology for conformational dynamics characterization, it is inevitable to have false positive or false negative identification. Some fragments cannot retain the HDX information, such as peptides which are not covered by the HDX-MS experiment or the residues belongs to the first two residues of each peptide. If the structural dynamics is outside the time window of deuterium labeling, the HDX-MS would miss the protein’s structure perturbation. Besides, the sequence resolution of HDX-MS data is limited to the size of proteolytic peptides. If the peptide covered more than one epitope, it is hard to pinpoint the specific residue. HDX-MS can also detect allosteric effects upon binding to a ligand for a signal propagation (Hamuro 2021).

Thus, close manual supervision of the deuteration level remains crucial after the automatical calculation by the software. Besides, the basic knowledge about protein structure and function facilitates researchers to understand which regions of protein participate in conformation changes.

## RESULT AND DISCUSSION

To assess the quality of the purified Hsp90N protein, SDS-PAGE was performed. As shown in Fig. 2C, the molecular mass of the Hsp90N protein was estimated to be approximately 28.3 kDa. The single band in SDS-PAGE gel indicated that the Hsp90N protein was of high purity. The result from MALDI-TOF-MS further confirmed that the Hsp90N protein had an exact molecular mass of 28.3 kDa (Fig. 2D). As the Hsp90N protein was purified by Ni-NTA affinity chromatography with salt gradient (Block *et al.* 2009), there was no detergent contained either. Taken together, the Hsp90N protein sample was of high purity, devoid of any detectable contaminants, and suitable for downstream analysis.

**Figure 3:**
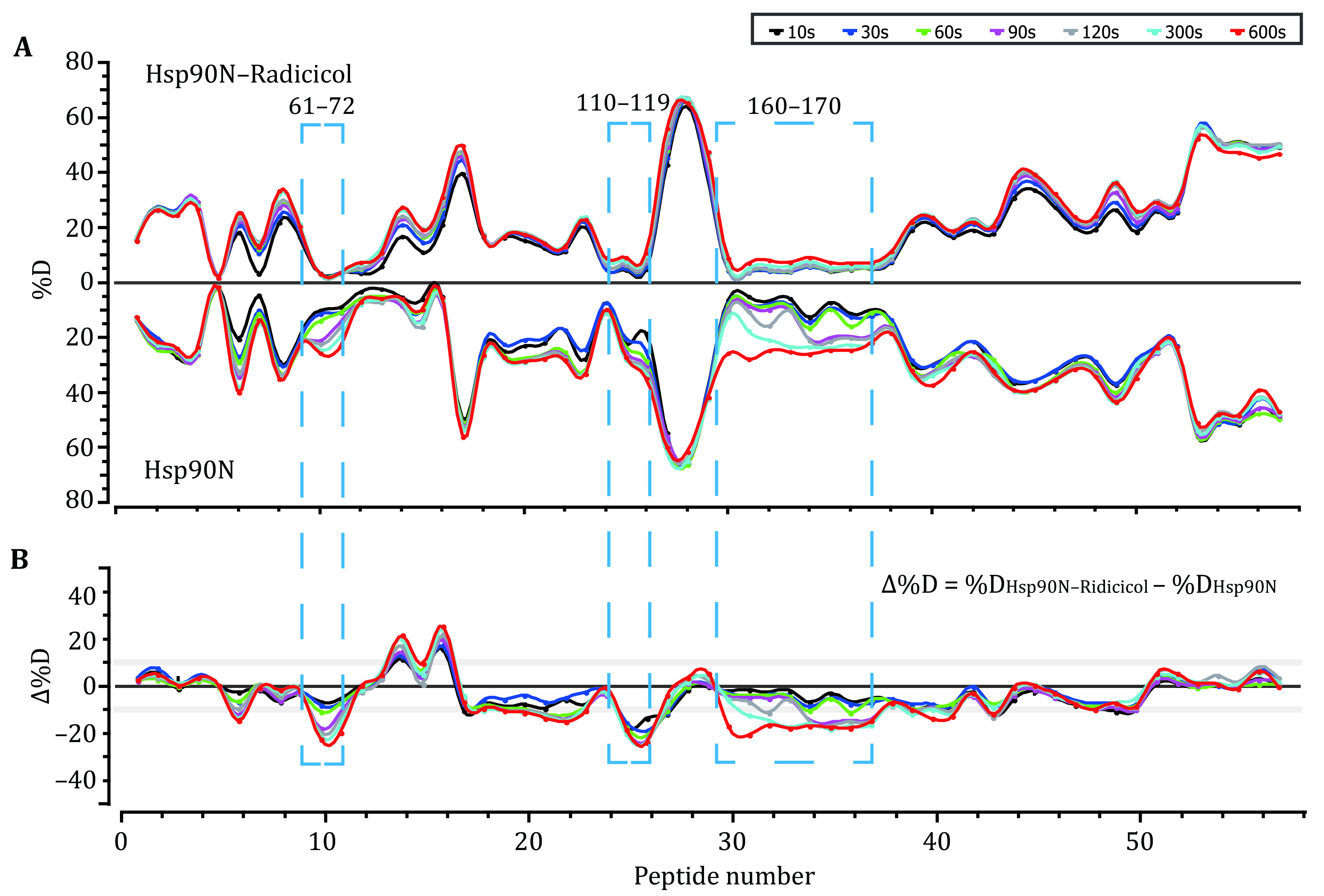
HDX kinetics comparison of the Hsp90N–Ridicicol complex and Hsp90 protein at the global peptide level. **A** Butterfly plot of the HDX kinetics of Hsp90N–Ridicicol complex (top) and Hsp90 protein (bottom). The *y*-axis is the deuteration percent of individual peptides. **B** Residual plot of individual peptides from the Hsp90N–Ridicicol complex and the Hsp90 protein. The *y*-axis is the deuteration percent difference of individual peptides calculated by equation (*i*.*e*., ∆%D = %D_Hsp90N–Ridicicol_ – %D_Hsp90N_) The gray lines at *y*-axis represent ±10% threshold for identifying significant differences between the Hsp90N–Ridicicol complex and the Hsp90 protein. Each data point is an average of three experiments. The black, dark blue, green, magenta, grey, and light blue lines correspond to data acquired at 10, 30, 60, 90, 120, 300, and 600 s of deuterium labeling, respectively, for both samples

**Figure 4:**
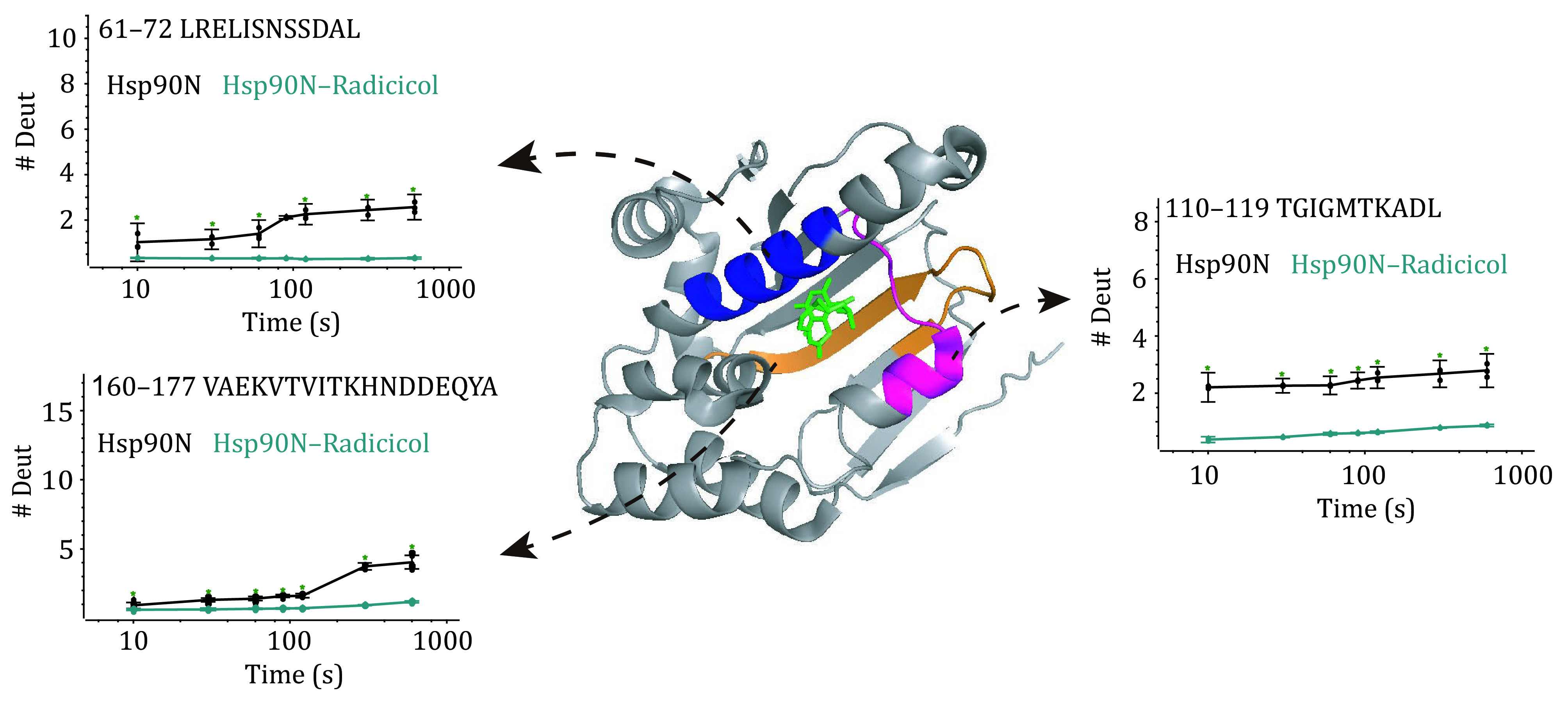
Comparison of the HDX kinetics for three peptides. The crystal structure of the Hsp90N is shown in the middle (PDB: 4EGK). The peptide LRELISNSSDAL (residues from 61 to 72) is indicated in blue. The peptide TGIGMTKADL (residues from 110 to 199) is indicated in magenta. The peptide VAEKVTVITKHNDDEQYA (residues from 160 to 177) is indicated in orange. The regions without significant deuteration change upon Ridicicol binding are mapped onto the structure of the Hsp90N protein in green. Ridicicol at the ligand binding pocket is shown as sticks. Black, the Hsp90N protein, cyan, Hsp90N–Ridicicol complex. These traces are an average of three replicate with error bars at each time point

To understand the impact of the small molecule on the higher order structure of the Hsp90N protein, a side-by-side HDX comparison of the Hsp90N–Radicicol complex and Hsp90N protein was performed. Deuterium uptake was assessed over seven-time points from 0 to 600 s, as described earlier. A total of 106 peptides were obtained corresponding to a sequence coverage of 83% of the primary sequence of the Hsp90N protein. The HDX kinetics of these peptides from the Hsp90N–Radicicol complex and Hsp90N protein were illustrated in Fig. 3A as a butterfly plot. In general, the HDX profiles of the Hsp90N–Radicicol complex and the Hsp90N protein were very similar. For the purpose of comparing the difference in deuteration level, a plot of mass difference (∆D) for each peptide was drawn as Fig. 3B. To minimize measurement error, a significance threshold of ±10% deuteration level was used (Pan *et al.* 2014, 2015). For each time point, if the deuteration level difference of a peptide from two samples was more than 10%, the peptide indeed made sense. As shown in Fig. 4, peptides around the sequences of "LRELISNSSDAL" (residues from 61 to 72), "TGIGMTKADL" (residues from 110 to 199), and “VAEKVTVITKHNDDEQYA” (residues from 160 to 177) exhibited ∆D values above the significance threshold. In the presence of Radicicol, these peptides showed decreased deuterium uptake, especially the peptide "110–119". After deuterium labeling, the deuterium level of this peptide altered fast (*i*.*e*., 10 s), while others started to change after 300 s. This could be caused by the D93 which can form a hydrogen bond network while Radicicol interacts with the Hsp90N protein (Prodromou *et al.* 1997; Stebbins *et al.* 1997). The correlation of the peptide deuterium uptake difference with the protein structure could be visualized via the crystal structure mapping. Regions of flexibility with lower deuterium exchange upon Ridicicol binding were shown in blue (peptide 61–72), magenta (peptide 110–119), and orange (peptide 160–177). The HDX kinetics can also be shown as a heat map (supplementary Fig. S2). Overall, the experimental results are consistent with previous reports (Ali *et al.* 2006; Chandramohan *et al.* 2016). Our HDX-MS experiment can sensitively probe the higher order structure perturbation attributed to Radicicol binding to the Hsp90N protein.

**Figure 5:**
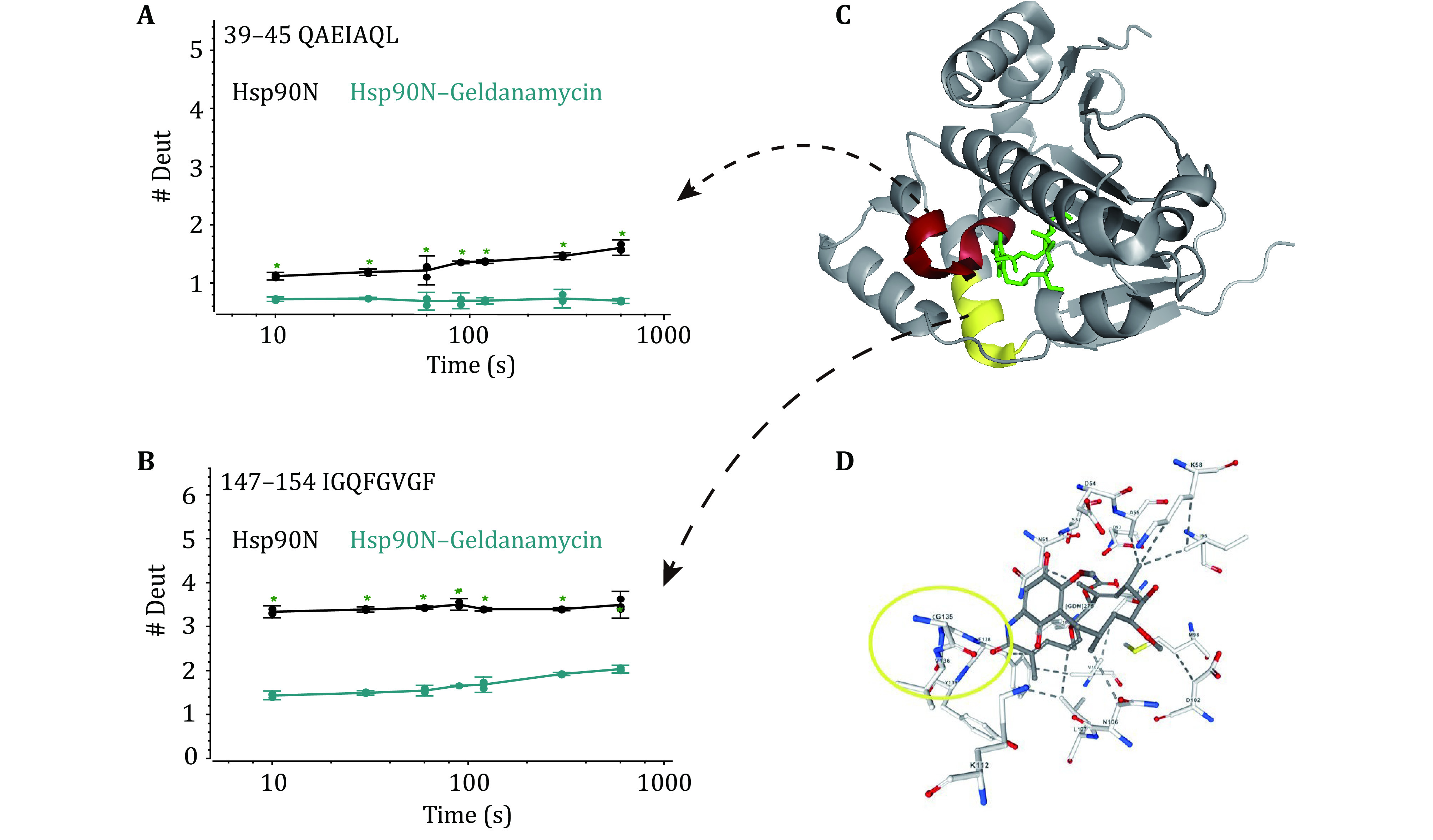
HDX kinetics comparison of the Hsp90N–Geldanamycin complex and Hsp90N protein. The deuterium uptake plots of the peptide QAEIAQL (residues from 39 to 45) (**A**) and the peptide IGQFGVGF (residues from 147 to 154) (**B**). **C** The crystal structure of the Hsp90N (PDB: 4XDM). **D** Backbone amide hydrogen bonding network of the Hsp90N

As the HDX-MS experiment is highly sensitive to experimental conditions, to prove the reproducibility of our HDX-MS protocol, we further characterized the conformational dynamics of the Hsp90N protein upon Geldanamycin binding. Geldanamycin, like Radicicol, exerts its *in vivo* function (*e*.*g*., antiproliferative and antitumor) by binding to the Hsp90N protein. Our HDX-MS experiment revealed that peptides around the sequences of "QAEIAQL" (residues from 39 to 45) and "IGQFGVGF" (residues from 147 to 154) showed decreased deuterium uptake (Fig. 5C and 5D) in response to the binding of Geldanamycin. To better understand the relationship between structure and function, we highlighted these sequences in the crystal structure of Hsp90N (Fig. 5B). It is apparent that G135, V136, and F138 form a stable hydrogen-bonding network (Fig. 5A). The side chain of D40 can also form a hydrogen bond with the amino group of K44 (Stebbins *et al.* 1997). Geldanamycin can disrupt the conformation of the Hsp90N protein in these regions. This is consistent with our HDX-MS experimental result as the peptide “39–45” and the peptide “147–154” showed lower deuterium uptake levels (supplementary Fig. S3).

At present, there are several available HDX-MS related protocols for studying protein structure and dynamics. For example, Lento *et al*. compared the deuterium profiles of native and phopho-tau (Lento *et al.* 2017). Hentze *et al*. revealed the interaction interface of the Hsp90 protein and the Sti 1 protein (Hentze and Mayer 2013). Our protocol is the first available to study protein–small molecule interaction. We characterized the conformational dynamics of the Hsp90N protein upon Radicicol or Geldanamycin binding. Besides the detailed protocol, we also briefly explain the fundamental theory of HDX-MS which would facilitate the novices to quickly start the experiments. Overall, HDX-MS can understand how protein structure and dynamics are influenced by small molecule binding. Our experimental protocol is robust, sensitive, and easy to use for novices.

### Limitations of the study

The sequence coverage of the Hsp90 protein was 83%. With the relatively low sequence coverage, it is hard to get single amide resolution data. As the related experimental parameters were already optimized, we thought the main reason accounting for this phenomenon was the MS equipment. Our protocol adopted Orbitrap Elite hybrid ion trap-orbitrap MS to detect peptic peptides. We can adopt advanced MS equipment such as Orbitrap Fusion to further increase the protein’s sequence coverage. Although we use Thermo's instrument, the HDX-MS protocol is applicable to MS produced by different manufacturers such as Waters (Houde *et al*. 2009), Agilent (Arora *et al.* 2015), *et al*.

## Conflict of interest

Qian Meng, Yuan-Li Song, Chen Zhou, Han He, Naixia Zhang and Hu Zhou declare that they have no conflict of interest.
